# Profiles of cytokine and chemokine gene expression in human pulmonary epithelial cells induced by human and avian influenza viruses

**DOI:** 10.1186/1743-422X-7-344

**Published:** 2010-11-26

**Authors:** WY Lam, Apple CM Yeung, Ida MT Chu, Paul KS Chan

**Affiliations:** 1 Department of Microbiology, The Chinese University of Hong Kong, New Territories, Hong Kong Special Administration Region, People's Republic of China; 2 Stanley Ho Centre for Emerging Infectious Diseases, The Chinese University of Hong Kong, New Territories, Hong Kong Special Administration Region, People's Republic of China

## Abstract

Influenza pandemic remains a serious threat to human health. In this study, the repertoire of host cellular cytokine and chemokine responses to infections with highly pathogenic avian influenza H5N1, low pathogenicity avian influenza H9N2 and seasonal human influenza H1N1 were compared using an *in vitro *system based on human pulmonary epithelial cells. The results showed that H5N1 was more potent than H9N2 and H1N1 in inducing CXCL-10/IP-10, TNF-alpha and CCL-5/RANTES. The cytokine/chemokine profiles for H9N2, in general, resembled those of H1N1. Of interest, only H1N1, but none of the avian subtypes examined could induce a persistent elevation of the immune-regulatory cytokine - TGF-β2. The differential expression of cytokines/chemokines following infection with different influenza viruses could be a key determinant for clinical outcome. The potential of using these cytokines/chemokines as prognostic markers or targets of therapy is worth exploring.

## Background

Avian influenza viruses (AIV) are classified into two pathotypes. The highly pathogenic type (HPAIV) causes severe disease with a high mortality rate, whereas the low pathogenic type (LPAIV) causes asymptomatic infection or a mild disease [1,2]. Human infection with HPAIV H5N1 was first detected in Hong Kong in 1997 [3-5]. As at July 2009, more than 400 human infections have been reported to the World health Organization (WHO), and with an average case fatality rate of greater than 60% (WHO 2010). Hypercytokinaemia was consistently reported from patients with fatal H5N1 infection [4,6-9].

Influenza viruses of the H9 subtype have been widely circulated in the world since their first detection from turkeys in Wisconsin in 1966 [10]. H9N2 viruses had caused disease outbreaks in chicken, ducks and pigs in many parts of the world including China, Germany, Hong Kong, Indonesia, Iran, Ireland, Israel, Italy, Jordan, Pakistan, Saudi Arabia, South Africa, South Korea, UAE, and USA in recent years [11-18]. In 1999 and 2003, self-limiting mild human infections with LPAIV H9N2 viruses were recorded in Hong Kong [19]. Some avian H9 viruses have acquired receptor binding characteristics typical of human strains, which may increase the potential for reassortment in both human and swine respiratory tracts [20-22].

The respiratory epithelial cells are the primary targets for HPAIV and LPAIV infections [23-25]. In response to HPAIV and LPAIV, these cells are likely to play a critical role in inflammatory response, and in the initiation of innate and subsequently adaptive immune responses [3,25-29]. Recently, it has been reported that HPAIV H5N1 infection of epithelial cells induce the expression of several proinflammatory cytokines and chemokines both *in vitro *and *in vivo*, which could be linked to the consequence of fatal hypercytokinemia [8,30-32].

The biological basis accounting for the difference in disease severity among different avian influenza virus infections in humans remains unknown. In this study, we compared the effect of different avian and human influenza subtypes on the induction of cytokine and chemokine expression using an *in vitro *model.

## Results

### Influenza virus replication

**Figure 1:**
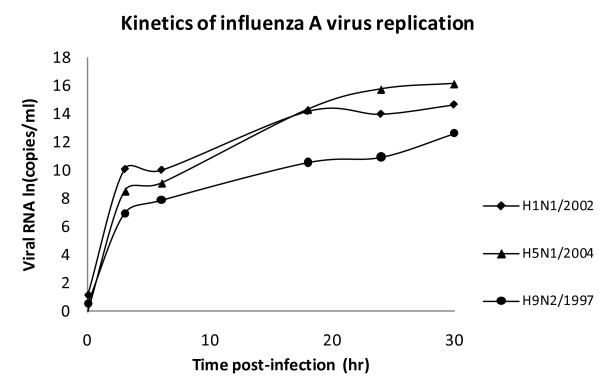
**Kinetics of replication of different subtypes of influenza A virus in NCI-H292 cells**. NCI-H292 cells were infected with different influenza virus subtypes: H1 - H1N1/2002, H5 - H5N1/2004, and H9 - H9N2/1997 with an m.o.i. of 1. Plasmid copy number expressed in natural logarithm (ln).

A similar rate of change in viral RNA copy numbers following the inoculation of human and avian viruses was observed for H1N1/2002 and H5N1/2004 indicating that these viruses replicated with a similar kinetic in the cell culture system (Figure 1). H9N2/1997 virus was found to replicate at a lower rate than the other two subtypes. All the virus subtypes reached the plateau level within 6 hours post-infection, and then increased steadily (Figure 1).

### Cytokine/chemokine mRNA expression during the early phase of viral infection

**Figure 2:**
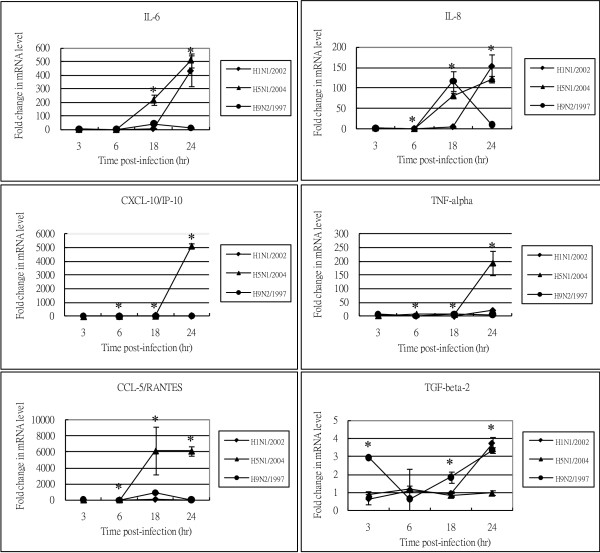
**Cytokine and chemokine mRNA levels at various time points post-infection**. NCI-H292 cells were infected with influenza A virus subtypes: H1N1/2002, H5N1/2004, and H9N2/1997 viruses at m.o.i. = 1. Real-time PCR were used to quantitify the mRNA levels and fold-changes were calculated by ΔΔ^CT ^method as compared with non-infection cell control and using endogeneous actin mRNA level for normalization. Each point on the graph represents the mean fold change in gene expression relative to NI - non-infected cells level ± SE (p* < 0.05).

The quantitative real-time RT-PCR results showed that during the early phase of infection (i.e. 3 and 6 hours post-infection), there was an induction of pro-inflammatory cytokines/chemokines. At 3 hours post-H5N1/2004 infection, there were 2-5 folds increase in the expression of TNF-α and CCL-5/RANTES. At 6 hours post-H5N1/2004 infection, there were marked increase in the expression of CXCL-10/IP-10 and CCL-5/RANTES (60-120 folds); while there were only relatively minor increase in IL-6 and IL-8 expression (2-10 folds) (Figure 2, Table 1).

**Table 1:** Cytokine/chemokine mRNA expression during the early phase of viral infection

Fold-changes	TNF-α	CCL-5/RANTES	CXCL-10/IP-10	IL-6	IL-8	TGF-β2
H5N1/2004	2-5	2-5	60 - 120	2-10	2-10	1

H9N2/1997	5-25	5-25	5-25	5	5	3

H1N1/2002	< 10	< 10	< 10	< 10	< 10	< 10

Similarly for H9N2/1997 infection, there was 5-25 folds increase in the transcription of CCL-5/RANTES, TNF-α, and CXCL-10/IP-10 mRNA at 3 hours post-infection. At 6 hours post-infection, the level of previously elevated cytokines/chemokines still remained at several folds of induction. In contrast to the prominent induction of cytokines/chemokines observed for avian subtypes, the induction by human subtype H1N1 was always below 10 folds during the early phase of infection.

In summary, up-regulation of mRNA for TNF-α, CCL-5/RANTES, and CXCL-10/IP-10 was found to be more prominent during the early phase of infection with H5N1/2004 and H9N2/1997 viruses than those induced by H1N1/2002 virus.

### Cytokine/chemokine mRNA expression during the late phase of infection

At the late phase of H5N1 infection, more intense induction of cytokine/chemokine expression was observed. At 18 hours post-infection, IL-6, CCL-5/RANTES and CXCL-10/IP-10 were highly expressed (12 to >1000 folds) in H5N1/2004 infection. Meanwhile, TNF-α and IL-8 were expressed at >10 folds in H5N1/2004 infection. At 24 hours post-infection, the previously elevated cytokines/chemokines were still remained at high levels. As for H5N1/2004 infection, CCL-5/RANTES and CXCL-10/IP-10 were found to be induced to >1000 folds; whereas TNF-α and IL-8 were expressed at 200-300 folds. (Figure 2, Table 2).

**Table 2:** Cytokine/chemokine mRNA expression during the late phase of viral infection

Fold-changes	TNF-α	CCL-5/RANTES	CXCL-10/IP-10	IL-6	IL-8	TGF-β2
H5N1/2004	200-300	12 - >1000	12- >1000	12 - >1000	200-300	1

H9N2/1997	5-25	1000	16-116	16-116	16-116	3

H1N1/2002	4-450	4-450	4-450	4-450	4-450	4-450

Similarly, at 18 hours post-H9N2/1997 infection, CCL-5/RANTES mRNA expression was found to be induced by nearly 1000 folds; while CXCL-10/IP-10, IL-6, and IL-8 were found to be up-regulated by 16-116 folds.

Although no significant cytokine/chemokine induction was observed during the early phase of H1N1 infection; IP-10/CXCL-10, TNF-α, TGF-β2, CCL-5/RANTES, IL-8, and IL-6 were found to be 4-450 folds induced during the late phase of infection (Figure 2, Table 2).

In summary, at the late phase of infection (i.e. 18 and 24 hours post-infection), TNF-α, IL-6, IL-8, CCL-5/RANTES and CXCL-10/IP-10 mRNA remained at high levels for H5N1/2004 and H9N2/1997; which were in contrast to those observed for H1N1/2002 (Figure 2, Table 2). The up-regulation of these mRNA was more prominent in H5N1/2004 infected cells, and the maximal up-regulation of these mRNA in H5N1/2004 infection occurred at 24 hours post-infection (Figure 2). Overall, the intensity of cytokine/chemokine mRNA induction in human H1N1/2002 was much lower than that observed in avian H5N1 and H9N2. Interestingly, the TGF-β2 mRNA was found to be up-regulated for H1N1/2002 and H9N2/1997, but not for H5N1/2004 (Figure 2, Table 2).

### Cytokine/chemokine protein profiles following infection

**Figure 3:**
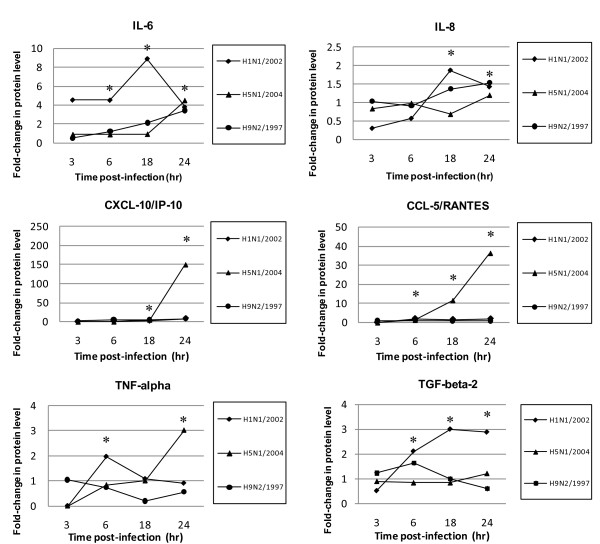
**Cytokine and chemokine protein levels at various time-points post-infection**. NCI-H292 cells were infected with influenza A virus subtypes: H1N1/2002, H5N1/2004, and H9N2/1997 at m.o.i. = 1. Graphs showing the fold-changes of protein levels as compared with non-infected cell control ± SE (p* < 0.05) at the corresponding time-point post-infection.

To verify whether changes at the mRNA level were translated to protein level, the protein concentrations of cytokines/chemokines in cell culture supernatants were measured (Figure 3, Table 3). The results showed that the epithelial cells secreted high amounts of IL-6, IL-8, CXCL-10/IP-10, and CCL-5/RANTES in response to influenza virus infections. In H5N1/2004 and H9N2/1997 infections, IL-6 was induced to a high level at 24 hours post-infection (4 and 3 folds, respectively); while H1N1/2002 induced a high level of IL-6 (about 8 folds) at 18 hours post-infection. The expression profile for IL-8 and CXCL-10/IP-10 was similar to IL-6. In H5N1/2004 infection, induction of cytokine/chemokine was prominent at the late phase (24 hours post-infection). H5N1/2004 showed 150 folds of induction for CXCL-10/IP-10. IL-6 and CXCL-10/IP-10 were induced in H9N2/1997 infections; but at relatively lower fold-changes than those of H5N1 throughout the time course examined (Figure 3, Table 3). The highest level of induction (36 folds) for CCL-5/RANTES was observed at the late phase of H5N1/2004 infection (18-24 hours) (Figure 3, Table 3). In general, H5N1/2004 showed a higher capacity in inducing CXCL-10/IP-10 and CCL-5/RANTES as compared with that of H1N1 and H9N2; and the effects were more prominent at the late phase of infection, particularly at 24 hours post-infection. Also, the cytokine/chemokine protein levels correlated with the corresponding mRNA transcription levels for all the subtypes except that there were some deviations at the late phase of H1N1 infection.

**Table 3:** Cytokine/chemokine protein profiles following viral infection

Fold-changes	TNF-α	CCL-5/RANTES	CXCL-10/IP-10	IL-6	IL-8	TGF-β2
H5N1/2004	3	36	150	4	1.3	1

H9N2/1997	1.2	3	12	3	1.6	1.5

H1N1/2002	1	2	10	8	1.4	3

TNF-α was induced by all subtypes beginning at the early phase of infection. A 3-fold increase in TNF-α secretion in late H5N1/2004 infection was also observed, and these results correlated with the TNF-α mRNA levels.

No induction in TGF-β2 level for H5N1/2004 was observed throughout the time course examined. The TGF-β2 level of H9N2/1997 only showed a transient elevation at 6 hours post-infection. In contrast, the elevation of TGF-β2 level of H1N1/2002 was sustained and increased with time reaching 2-3 folds at 18 and 24 hours post-infection (Figure 3, Table 3).

## Discussion

Lung epithelial cells are the key target of influenza viruses [33,34]. However, to date, most studies on influenza virus-induced inflammatory cytokines have been based on macrophages and monocytes infected *in vitro *or *in vivo *[35-37]. The mechanism concerning bronchial infiltration of inflammatory cells, particularly lymphocytes and eosinophils, and the subsequent hyperresponsiveness of the bronchial wall induced by viral infection remains unclear [38].

Due to the fact that HPAIV and LPAIV infection can cause a different degree of immune response, we hypothesized that the highly pathogenic properties of HPAIV may be caused by two determinants: firstly, the viruses have the ability to over-induce proinflammatory cytokines, for example, excessive activation of the pathogen detecting receptors, which may result in excessive secondary cytokine/chemokine response. Secondly, the viruses may directly or indirectly interfere with the balance of cytokine/chemokine production. For example, the feedback mechanism of cytokine/chemokine biosynthesis may be interrupted by the viral components.

Cytokines and chemokines generally function in an autocrine (on the producing cell itself) or paracrine (on nearby cells) manner. Cytokines released following infection can be classified broadly into "early" and "late" cytokines. In this study, the transcription levels of 6 cytokines/chemokines were delineated over the 24-hour period following virus inoculation. Recently, it has been found that the inflammatory response is played out over time in a reproducible and organized way after an initiating stimulus. It had been suggested that genes activated in mouse fibroblasts in response to the cytokine TNF-α could be categorized into roughly three groups, each with different induction kinetics [39]. These observations are in line with our findings that the cytokine/chemokine response profile varied with the time-course of infection. Our results showed that at the early phase of avian influenza virus infection, the transcription of TNF-α and IL-6 was induced. At the late phase of infection; induction of IL-8, CCL-5/RANTES, and CXCL-10/IP-10 occurred.

Although TNF-α was first noted for its role in killing tumor cells [40], it also has pleiotropic functions including inflammatory response and host resistance to pathogens [34,41]. TNF-α may activate nuclear factor-kB (NF-kB) by inducing the phosphorylation and degradation of inhibitory factor-kB (IkB) and leads to the translocation of NF-kB to the nucleus where it can bind to specific-binding sites of the relevant promoters. It has been reported that NF-kB regulates many kinds of genes and plays a crucial role in inflammatory diseases [39,42]. Subsequent binding of NF-kB to the CCL-5/RANTES promoter has also been reported [43,44]. In line with this, we also observed an induction of CCL-5/RANTES in avian influenza infection, which may then attract monocytes, eosinophils, basophils, and CD4+ T cells [45]. CCL-5/RANTES production from bronchial epithelial cells contributes to infiltration of inflammatory cells in the airway during viral infection. The other chemokine, CXCL-10/IP-10, found upregulated by avian influenza viruses is a macrophage chemo-attractant that mediates inflammatory response by further recruitment of circulating leukocytes into the inflamed tissues [25]. In addition, IL-8 is also a potent chemo-attractant and stimulus of neutrophils. It plays a pivotal role in inflammatory diseases. It is also well known that IL-6 plays an important role in the stimulation of B lymphocytes for antibody production. TNF-α together with IL-6 may boost proliferation and differentiation of B cells, and proliferation of T cells. As a result, all these TNF-α activated mediators could contribute to the infiltration of inflammatory cells into the influenza infected respiratory tract.

Our results showed that H5N1 was a potent inducer of CXCL-10/IP-10 and CCL-5/RANTES. The induction of these cytokines/chemokines might be initially achieved by a trace amount of TNF-α secretion as detected during the initial phase of infection. Therefore, initial TNF-α secretion might be critical to account for the high pathogenicity of H5N1 infection.

Although seasonal influenza A/H3N2 has been more prevalent over the last 10 years, and there is evidence that it is more virulent in humans [46-48], we chose H1N1 because of its lower pathogenicity and therefore a better reference for comparison with the highly pathogenic H5N1 virus.

Another important aspect of balancing cytokine/chemokine production is the role of the anti-inflammatory mediators. Accordingly, the secretion of a well-known anti-inflammatory cytokine/chemokine, TGF-β2, was measured in this study. Our data showed that H1N1 induced the highest transcription of TGF-β2 mRNA, and was the only subtype that could induce a sustained increase in TGF-β2 at protein level. Since TGF-β can act as both an immunosuppressive agent and a potent proinflammatory molecule through its ability to attract and regulate inflammatory molecules, it plays a vital role in T-cell inhibition. Furthermore, it has been reported that TGF-β2 inhibits Th1 cytokine-mediated induction of CCL-5/RANTES, CCL-3/MIP-1α, CCL-4/MIP-1β, CCL-9/MIP-1γ, CXCL-2/MIP-2, CXCL-10/IP-10, and CCL-2/MCP-1 [49]. It has also been found that in real bronchial environments, TGF-β mediates cross-talk between alveolar macrophages and epithelial cells [50]. We therefore speculate that, inside the lungs, the activated inflammatory cascade launches a quick antimicrobial reaction and directs adaptive immunity to mount a protective response. The pro-inflammatory response is tightly controlled by mediators, such as TGF-β, to protect the easily damageable lung tissue from destructive side effects associated with virus induced inflammation. Our speculation coincides with other studies which demonstrated that highly pathogenic H5N1 virus infection in mice model could cause a down-regulation of TGF-β secretion which resulted in more severe and widespread lesions [51]. These may also account for the difference in pathogenicity of different AIV strains [52,53].

Recently, a concept of organ-specific and graded immune responses was proposed by Eyal Raz [54]. According to this concept, each organ senses infectious dangers in a specific way, and the organ-specific physiology modulates and instructs the local immune response. It has been reported that there is a unique regulatory mechanism of toll-like receptor (TLR) activation pathways that is intrinsic to the lungs. Bronchial epithelial cells modulate the activation of monocytes, macrophages, dendritic cells (DC), and T lymphocytes; thus contributing to the generation of a specific bronchial homeostatic microenvironment that affects the way in which the body copes with the viruses. This homeostatic "circuit" can inhibit excessive inflammatory response in lung tissues [55]. Therefore, the exact regulatory role of this cytokine - TGF-β2, and its association with TLR activation in the initiation, progression, and resolution of immune response during infection with influenza viruses with different pathogenicity is worthy for further study.

## Conclusion

There are qualitative and quantitative differences in the profiles of cytokines/chemokines induced by influenza viruses of different pathogenicity. H5N1 was a more potent inducer of inflammatory cytokines/chemokines; particularly TNF-α, CXCL-10/IP-10, and CCL-5/RANTES in lung epithelial cells. In contrast, H1N1 showed more potent induction of the anti-inflammatory cytokine - TGF-β2.

## Materials and methods

### Virus isolates

The influenza A H5N1 virus (A/Thai/KAN1/2004) (H5N1/2004) was isolated from a patient with fatal infection in Thailand in 2004. The H9N2 isolate (A/Duck/Hong Kong/Y280/1997) (H9N2/1997) was collected in Hong Kong and was closely related to those found from human H9 infections. These isolates represented avian influenza of high and low pathogenicity. To serve as a comparison, a human H1N1 strain isolated in 2002 - (A/HongKong/CUHK-13003/2002) (H1N1/2002) was included.

### Cell cultures

The bronchial epithelial cells, NCI-H292 (ATCC, CRL-1848, Rockville, MD, USA), derived from human lung mucoepidermoid carcinoma were grown as monolayers in RPMI-1640 medium (Invitrogen, Carlsbad, CA) supplemented with 10% fetal bovine serum (FBS), 100 U/ml penicillin and 100 μg/ml streptomycin (all from Gibco, Life Technology, Rockville, Md., USA) at 37°C in a 5% CO_2 _incubator. Mandin-Darby canine kidney (MDCK) cells were used for growing stocks of influenza virus isolates. MDCK cells were grown and maintained in Eagles Minimal Essential Media (MEM) containing 2% FBS, 100 U/ml penicillin and 100 μg/ml streptomycin (all from Gibco, Life Technology).

### Infection of cell culture with influenza A viruses

NCI-H292 cells were grown to confluence in sterile T75 tissue culture flasks for the inoculation of virus isolate at a multiplicity of infection (m.o.i.) of one. After 1 hour of adsorption, the virus was removed and 2 ml of fresh RPMI-1640 media with 2% FBS, 100 U/ml penicillin, 100 μg/ml streptomycin and 1 μg/ml L-1-tosylamido-2-phenylethyl chloromethyl ketone (TPCK)-treated trypsin (all from Gibco, Life Technology) was added, and incubated at 37°C in 5% CO_2 _humidified air.

### Harvest of host cell RNA

The infected cell cultures and the non-infected controls were harvested at 3, 6, 18 and 24 hours after virus inoculation. Total RNA was extracted from the cell lysate using TRIzol-total RNA extraction kit (Invitrogen) according to the manufacturer's procedures. The extracted RNA was eluted in 30 μl of nuclease-free water, and stored in aliquots at -80°C until used. In order to avoid contamination with genomic DNA, the extracted preparation was treated with DNA-Free DNase (Invitrogen) according to the manufacturer's instructions. The quality of RNA in the extracted preparation was analyzed by measuring optical density at 260/280 nm with the NanoDrop ND-1000 spectrophotometer (NanoDrop Technologies).

### Quantitation of viral replication

The cDNA was synthesized from previously prepared mRNA with poly(dT) primers and SuperScript III reverse transcriptase (Invitrogen). Quantitative *Taq*man real-time PCR assay was used to measure the level virus produced in cell culture supernatant. Specific primers amplifying the conserved region of the M gene of influenza A viruses were used, and quantitative real-time PCR analysis was performed with an ABI PRISM 7700 sequence detection system (Applied Biosystems, Foster City, CA). Preparations with known copy numbers of plasmids cloned with the M gene were used for standard curve construction. The β-actin gene was used as an endogenous control for normalization [56].

### Cytokine/chemokine mRNA expression profile

Total RNA extracted from cell cultures was reversely transcripted to cDNA using the poly(dT) primers and Superscript III reverse transcriptase (Invitrogen), and quantified by real-time PCR. The sense and antisense primers used in real-time PCR for measuring the cytokines/chemokines (CCL-5/RANTES, CXCL-10/IP-10, IL-6, IL-8, TNF-α, TGF-β2) are listed in Table 4. The real-time PCR reactions were performed in triplicate using the SYBER Green PCR Master Mix (Applied Biosystems). The PCR conditions were 95 °C for 5 min, followed by 50 cycles of 95 °C for 30 sec, 55 °C for 30 sec, and 72 °C for 30 sec. The expression of β-actin gene was also quantified in a similar way for normalization. The comparative delta-delta C_T _method was used to analyze the results with the expression level of the respective gene at the corresponding time point in non-infected cells regarded as one [57,58].

**Table 4:** Primers used in real-time PCR assays.

Amplification target	Forward primer (5'-3')	Reverse primer (5'-3')
CCL-5/RANTES	CCCCATATTCCTCGGACACCACA	GTTGGCACACACTTGGCGGTTC

CXCL-10/IP-10	TCGAAGGCCATCAAGAATTT	GCTCCCCTCTGGTTTTAAGG

IL-6	ATTCTGCGCAGCTTTAAGGA	GAGGTGCCCATGCTACATTT

IL-8	TGTGCCTTGGTTTCTCCTTT	GCTTCCACATGTCCTCACAA

TGF-beta-2	CCAAAGGGTACAATGCCAAC	TAAGCTCAGGACCCTGCTGT

TNF-alpha	CCTGGGATTCAGGAATGTGT	AGGCCCCAGTTTGAATTCTT

Beta-actin	GCACGGCATCGTCACCAACT	CATCTTCTCGCGGTGGCCT

Primers for cytokine/chemokine detection and primers for actin detection.

### Quantification of cytokine/chemokine protein expression

Cell culture medium supernatant was collected at 0, 3, 6, 18, and 24 hours post-infection for the analysis of cytokine/chemokine expression. TNF-α, IL-6, IL-8, CXCL-10/IP-10, and CCL-5/RANTES were measured by the Cytometric Bead Array (CBA) Soluble Protein Flex Set system (BD™, San Jose, CA) using the BD FACSCalibur Flow Cytometer System (BD Biosciences) according to the manufacturer's instructions. The biologically active form of TGF-β2 was measured by enzyme-linked immunosorbent assay (Emax^® ^ImmunoAssay System, Promega, Madison, WI, USA) because a CBA system for this cytokine was not available.

## Competing interests

The authors declare that they have no competing interests.

## Authors' contributions

ACMY performed RT-PCR assays, flow-cytometry assays and IMTC participated in virus culture and virus isolation. WYL was responsible for experimental design, analyses and drafting of the manuscript. PKSC was responsible for design and supervision of the study. All authors read and approved the final manuscript.
